# AnVILWorkflow: A runnable workflow package for Cloud-implemented bioinformatics analysis pipelines

**DOI:** 10.21203/rs.3.rs-4370115/v1

**Published:** 2024-05-15

**Authors:** Sehyun Oh, Kai Gravel-Pucillo, Marcel Ramos, Sean Davis, Vince Carey, Martin Morgan, Levi Waldron

**Affiliations:** City University of New York School of Public Health; City University of New York School of Public Health; City University of New York School of Public Health; University of Colorado Anschutz School of Medicine; Harvard Medical School; Roswell Park Comprehensive Cancer Center; City University of New York School of Public Health

## Abstract

Advancements in sequencing technologies and the development of new data collection methods produce large volumes of biological data. The Genomic Data Science Analysis, Visualization, and Informatics Lab-space (AnVIL) provides a cloud-based platform for democratizing access to large-scale genomics data and analysis tools. However, utilizing the full capabilities of AnVIL can be challenging for researchers without extensive bioinformatics expertise, especially for executing complex workflows. Here we present the AnVILWorkflow R package, which enables the convenient execution of bioinformatics workflows hosted on AnVIL directly from an R environment. AnVILWorkflowsimplifies the setup of the cloud computing environment, input data formatting, workflow submission, and retrieval of results through intuitive functions. We demonstrate the utility of AnVILWorkflowfor three use cases: bulk RNA-seq analysis with Salmon, metagenomics analysis with bioBakery, and digital pathology image processing with PathML. The key features of AnVILWorkflow include user-friendly browsing of available data and workflows, seamless integration of R and non-R tools within a reproducible analysis pipeline, and accessibility to scalable computing resources without direct management overhead. While some limitations exist around workflow customization, AnVILWorkflowlowers the barrier to taking advantage of AnVIL’s resources, especially for exploratory analyses or bulk processing with established workflows. This empowers a broader community of researchers to leverage the latest genomics tools and datasets using familiar R syntax. This package is distributed through the Bioconductor project (https://bioconductor.org/packages/AnVILWorkflow), and the source code is available through GitHub (https://github.com/shbrief/AnVILWorkflow).

## 1. Introduction

The NHGRI’s Genomic Data Science *An*alysis, Visualization, and /nformatics Lab-space (AnVIL) consortium was launched in 2018, aiming to democratize genomics data[1]. AnVIL enables easy sharing of genomics data by organizing databases, bioinformatics pipelines for large-scale data processing, and interactive downstream analysis in one Cloud-based platform. AnVIL[2], also the name of the platform from the AnVIL project, implements the FAIR data-sharing philosophy and provides a graphical user interface (GUI, supported by Terra[3]), making it more accessible for researchers without programming backgrounds. However, a GUI tends to be less efficient and slower than a command line interface (CLI), especially for bulk analyses, still requires learning a new platform, and does not support version control and text-based workflows, often included as best practices for reproducible computational research[4]

Bioconductor’s *AnVIL* package is an AnVIL API wrapper that provides R-friendly, programming-based functionalities to leverage flexible and scalable cloud-based resources implemented in the AnVIL platform. With the *AnVIL* package, users can easily access workflows, data, and Cloud-based computing resources managed by AnVIL. However, the AnVIL package is not customized for workflow execution tasks. Instead, *AnVIL* covers all the resources related to the AnVIL platform, such as interaction with the repository for Docker-based genomic analysis tools and workflows (Dockstore[5]), leveraging cloud resources (Leonardo[6]), and data search and digestion (Gen3[7]). Many *AnVIL* functions also expose API commands directly, requiring a deep understanding of the underlying *AnVIL* workspace structures and data models to use for workflow execution. Also, it is a general package without individual support on any workspace and provides no metadata curation. Because the majority of Bioconductor users focus on data analysis, a convenient R-friendly way of accessing and utilizing AnVIL resources is needed. Here, we present the *AnVILWorkflow* package to meet this need. *AnVILWorkflow* package is a convenient, fit-for-purpose wrapper around the *AnVIL* package with the following features optimized for workflow execution:

- Intuitive function names and returned values
- Support workflow-specific documentations
- Enable to set up a Cloud environment with a single function call
- Return error messages that are easy to interpret and actionable
- Essential metadata curation for more efficient data browsing

Users can apply *AnVILWorkflow* on any workspace they can access, including 347 public workspaces (snapshot on 8.28.23) available to anyone with an AnVIL account. We present the three use cases where we ran non-R-based bioinformatics analysis tools using conventional R syntax: *Salmon[8], bioBakery[9]* and *PathML[10]. Salmon* is a widely used RNA sequencing analysis tool for quantifying the expression of transcripts and is based on the command-line interface. Its downstream analysis involves many R/Bioconductor packages, such as *DESeq2, edgeR*, and *limma. bioBakery* is a widely used whole metagenomic shotgun (WMS) sequencing data analysis environment, mainly relying on Python. *PathML* is a general-purpose research toolkit for computational pathology, including many functionalities in digital pathology data analysis, such as strain normalization, nucleus segmentation, and tissue detection. *PathML* takes raw image files and returns the processed image data in an hdf5 format for further downstream analysis, including machine learning methods.

## 2. Overview

**Figure 1:**
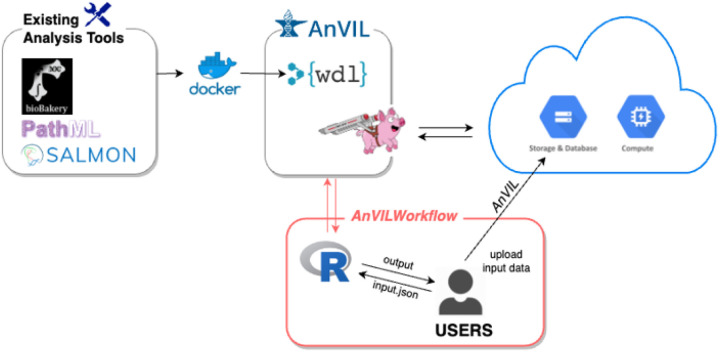
Overview of *AnVILWorkflow*package. AnVIL’s workflow description language (WDL) specified the runtime environment, which includes proper docker containers for existing analysis tools and computing resources. Cromwell, a scientific workflow execution and management system, runs WDL workflows on the cloud. *AnVILWorkflow* users can easily run established workflows developed by experts and utilize the cloud resources without configuring or taking maintenance responsibilities.

AnVIL provides comprehensive resources for biomedical data analysis, including data (e.g., genomics), workflows for bulk analysis, and interactive analysis apps (i.e., Galaxy, Jupyter Notebooks, and RStudio) under the workspace. Among them, workflows are often a limiting factor in bioinformatics analysis due to computing demands and bioinformatics expertise required. Thus, the *AnVILWorkflow* package makes the workflow-related resources from AnVIL more accessible and easier to use, especially for R users (Fig. 1).

While AnVIL manages workflow orchestration and workspace metadata and provides default setups simplifying decision-making for users, users still need to manage the storage of their data and cloud cost. Genomics data, especially their raw and intermediate forms, are very large, so data storage can be costly if the sample size increases. Storage costs incur and can be managed in two ways - storage itself and transfer. For example, using regional storage instead of multi-region, cleaning up intermediate results, and storing infrequently accessed data in low-cost storage (e.g., nearline or coldline storage from Google Cloud) can reduce per-sample costs. Analyzing data stored in one region using Virtual Machine (VM) compute resources in a different region incurs data transfer charges, so centralizing all storage and computing in a single region can be more cost-efficient by not only reducing the storage cost but also avoiding data transfer charges. Currently, the AnVIL workspaces use the us-central1 as a default region and any artifacts generated from the workflow execution, unless specified, are saved in the same-region bucket linked to the workspace. If users use the default region configured by AnVIL, bringing their data stored in the default region, us-central1, will save the data transfer charge. Additionally, open and controlled access genomic datasets hosted in AnVIL are stored in the us-multi-region, so there are no storage and transfer charges for users using the default workspace configuration. Downloading data to the user’s workstation or laptop is subject to charges, currently $0.08 to $0.12 per GB, depending on the amount of data[11] and geography of the transfer, and transfer from the US to another continent is more expensive than within the US transfer.

While browsing existing resources through *AnVILWorkflow is* free, running workflows charge computing costs. *AnVILWorkflow* is designed to use existing workflows which usually predefine computing resources optimized for the types of analyses, simplifying computing-related cost management. You can further reduce the run cost using call caching and preemptive instances. For example, if your workflow runs in fewer than 24 hours since a preemptible VM lasts 24 hours at most, you can save up to 80% by using preemptible VMs.

The cost management for a group of users can be efficiently managed through the AnVIL billing project. One billing account can be shared with others by simply adding email addresses under the billing project. The billing project offers details on each workspace, including workspace owner and spent reports, so we can easily identify ‘who’ uses ‘how much’ for ‘what’. In addition to the workspace-level expense reports, users can further enhance cost monitoring by configuring spending reporting[12]. This allows users to closely monitor the expenditure associated with each workflow execution.

## 3. Major functions

### Browse AnVIL resources.

The *AnVILBrowse* function allows users to browse AnVIL resources using keywords. This function runs instantaneously because the *AnVILWorkflow* package includes the snapshot of metadata on all the publicly accessible AnVIL workspaces and their workflows and data. It performs basic metadata harmonization, allowing more efficient browsing and filtering, such as selecting workspaces based on the study size or participants’ ages. Users can also browse non-public workspaces they have access to using the *getMetaTables* function; however, this process can take a while depending on the number of workspaces a user has access to.

### Run AnVIL workflows.

*AnVILWorkflow* package provides all the functionalities required to run workflows available in AnVIL from the local R session - from the environment setup to the output download. One prerequisite is to create an AnVIL account from the AnVIL web portal. AnVIL account provides two required inputs to run workflows remotely: 1) the email address associated with the user’s account and 2) the billing project name to cover the computing cost.

AnVIL-hosted workflows can be run using four main functions: setCloudEnv, cloneWorkspace, runWorkflow, and getOutput. The setCloudEnv function accepts the AnVIL account email and billing project name and sets up your local R environment ready to access AnVIL and Cloud-computing resources. The cloneWorkspace function creates the user’s copy of a ‘template’ workspace and the runWorkflow executes the workflow. The getOutput function can check the outputs from successfully executed workflows and download user-specified files to a local computer.

User input can be provided through the updatelnput function, which accepts two different forms of tables depending on the workflows - AnVIL’s data model or URLs pointing to data files stored in Google Cloud buckets. The input data formats are already specified in the workflow scripts (Workflow Description Language, WDL[13]). Other accessory functions are available to monitor submission progress (monitorWorkflow), stop submitted workflow (stopWorkflow), and get Dashboard content (getDashboard).

## 4. Use cases

The use cases demonstrated below include demo input data in the template workspaces, so the R scripts below can run the listed use cases from the local computer. Ready-to-run examples that can be used to test the process on the user’s own AnVIL account are available in the *AnVIL Workflow* package vignette. GATK best practice pipelines[14] are not demonstrated here, but they are also available as AnVIL workspaces.


## *Setup the account*
**setCloudEnv**(accountEmail = {AnVIL account email},
billingProjectName = {AnVIL billing project name})
## *Clone the workspace of your interest*
newName <- {Unique name for your copy of workspace}
**cloneWorkspace**(workspaceName = newName, templateName = templateName)
## *Run workflow*
**runWorkflow**(workspaceName = newName,
workflowName = {name of the workflow if there is more than one in the workspace of your interest})
## *Get workflow outputs*
**getOutput**(workspaceName = newName)


The main features of the demo workspaces and their workflow-specific input data preparation process are described below.

### 4.1. Bulk RNA sequencing data analysis

*Salmon* workflow uses AnVIL’s data model and requires four essential inputs - fastq1, fastq2, fasta, and *transcriptome index name*. This workflow can be easily applied to the consortium data hosted in AnVIL, which follows AnVIL’s data model. With the default runtime environment configured for this workflow (1 CPU, 2GB memory, and 10GB SSD disk), processing 16 demo samples (32 fastq files, ~1GB per file) took about 30 minutes and cost $0.12.

### 4.2. Whole metagenomic shotgun data analysis

*bioBakery* is a metagenome analysis environment composed of Python-based tools, reference databases, and command-line-based workflows. It processes raw shotgun sequencing data into microbial community feature profiles, summary reports, and figures[9]. *bioBakery’s* whole metagenome shotgun (wmgx) and visualization (wmgx_vis) workflows are implemented as an AnVIL workspace. The current version of the *AnVILWorkflow* supports *bioBakery*version 3[15]. While users can customize this workflow to a great degree, only six inputs are sufficient to run a standard, optimized version of this workflow. Those six inputs are:

- Name of the Trimmomatic adaptor type (for demo data, *NexteraPE)*
- Your project name
- Extension of input files (for demo data, .fastq.gz)
- A table of your sequencing file (fastq) names stored in the Google Cloud Storage bucket
- Input file identifier for paired-end sequencing (for demo data, _R1 and _R2)

The seven required databases are already linked to this workflow and nine additional optional inputs are available for further customization. Optional inputs are for workflow customization, such as bypassing functional profiling (default is false) and maximum memory usage for different tasks (default is 32GB for functional profiling by *HUMAnN*, 8GB for quality control by *Kneaddata*, and 24GB for taxonomic profiling by *MetaPhlAn)*. This workflow uses call caching and preemptive instances by default for cost efficiency. Processing six paired-end demo samples (mean file size ~ 380MB) with the optimized default setting without using preemptive instances took about 5 hours and cost around $6.50. With the preemptive instances, it can take longer but cost less. Compared to the existing options such as Nephele[16], *AnVILWorkflow* allows a programmatic approach and more flexible customization options.

### 3.3. Histopathology image processing using PathML

We implemented the hematoxylin-eosin (HE) stain normalization process of *PathML* as an AnVIL workspace. This workflow accepts an SVS file as input and returns original and normalized images as PNG files. There are two required inputs - Google Cloud Storage URI where the input SVS image file is stored and the sample name. Processing one publicly available image (CMU-1_Small_Region.svs, 1.8MB) [17] with the default runtime (4 CPU, 16GB memory) took about 8 minutes and cost $0.01. This simple but robust analysis setup can support clinical use cases, such as pathologists who process a large number of images in a short time, by offering guidance and cross-validation options.

## 5. Conclusions

The *AnVILWorkflow* package enables users to conduct complex and computationally intense analyses with minimal bioinformatics expertise, through well-established workflows within AnVIL and versatile cloud resources directly from standard laptops using the familiar R syntax. The major advantages *AnVILWorkflow* provides over the existing approaches include 1) a minimal entry barrier, negating the need for software installations, preparation of properly versioned reference data, or construction and oversight of workflows, 2) leveraging flexible cloud computing resources without the need to learn or handle them directly, 3) user-friendly functions that provide enhanced information, and 4) greatly improved reproducibility and interoperability by seamlessly linking multiple analysis steps, conducted in both R and non-R based tools, within a single R vignette. However, there are still some limitations. For instance, certain customizations of the workflows are limited or require a more profound understanding of the workflows. Despite not being inherently more costly than an in-house server, the pay-per-use structure requires careful planning and management. The absence of an integrated versioning system in AnVIL workspaces requires users to manually monitor new versions. In conclusion, *AnVILWorkflow* proves most advantages for analyzing a bulk of samples on relatively simple workflows (i.e., single-stage workflow procedure) or for exploratory data analysis for non-technical users, particularly when employing well-established analysis workflows.

## Data Availability

All data generated or analyzed during this study are included in this published article and available on the AnVIL platform (https://anvil.terra.bio/#). AnVILWorkflow package is distributed through the Bioconductor project (https://bioconductor.org/packages/AnVILWorkflow), and the source code is available through GitHub (https://github.com/shbrief/AnVILWorkflow).

## Abbreviations

AnVIL: Analysis, Visualization, and Informatics Lab-space
GUI: Graphical User Interface
CLI: Command Line Interface
WMS: whole metagenomic shotgun
VM: Virtual Machine
WDL: Workflow Description Language
HE: hematoxylin-eosin
HUMAnN: HMP Unified Metabolic Analysis Network
